# Clinical pharmacist-led antifungal drug utilization reviews in cancer care hospital: a prospective audit and feedback

**DOI:** 10.1093/jacamr/dlae184

**Published:** 2024-11-19

**Authors:** Zunaira Akbar, Muhammad Aamir, Zikria Saleem, Muhammad Rehan Khan, Omar Akhlaq Bhutta

**Affiliations:** Department of Pharmacy, The University of Lahore, Lahore, Pakistan; Riphah Institute of Pharmaceutical Sciences, Riphah International University, Lahore, Pakistan; Department of Pharmacy, The University of Lahore, Lahore, Pakistan; Faculty of Pharmacy, Bahauddin Zakariya University, Multan, Pakistan; Department of Pharmacy, Shaukat Khanum Memorial Cancer Hospital and Research Centre, Lahore, Pakistan; Department of Pharmacy, Shaukat Khanum Memorial Cancer Hospital and Research Centre, Lahore, Pakistan

## Abstract

**Background and objective:**

The global rise in systemic fungal infections and increased antifungal use underscores the need for pharmacist-led antifungal stewardship in oncology but data on such interventions are scarce. This study aimed to evaluate the clinical pharmacist-led antifungal drug utilization reviews for optimizing antifungal therapy in a specialized cancer care hospital.

**Patients and Method:**

This pharmacist-led prospective audit and feedback study evaluated 350 admitted patients with cancer who were prescribed systemic antifungals. Electronic medical records of the included patients were reviewed to evaluate pharmacist interventions. Data were entered and analysed through SPSS version 21.

**Result:**

Most of the patients were prescribed antifungals for suspected fungal infections (41.7%). Febrile neutropenia was present in 55.4% of patients. The most frequently isolated fungus was *C. albicans* (15.4%) followed by *C. tropicalis* (8.6%) and *A. flavus* (7.7%). The most frequently prescribed antifungal drug was voriconazole (38.8%) and amphotericin B (31.7%). Major pharmacist-led interventions were a change of IV antifungal therapy to an oral drug (18%), choice of drug therapy (17.4%) and dose reduction (16.9%). All the interventions made by the pharmacist were accepted by the AFS team (100%).

**Conclusion:**

Pharmacists play a crucial role in optimizing antifungal therapy by conducting drug utilization reviews and implementing targeted interventions. These interventions are beneficial for overall management of patients with cancer and improving the quality of antifungal prescribing.

## Introduction

The incidence of systemic fungal infections has been increased globally due to rise in immunocompromised patients afflicted with various diseases.^1^ In patients with cancer undergoing aggressive cytotoxic therapy, invasive fungal infections (IFI) are significant contributors to mortality.^2^ Up to 40% of individuals with haematological malignancies experience IFI, with Aspeirgillus and Caindida species collectively accounting for ∼95% of total infections.^3^ The integration of novel antifungal drugs into clinical settings carries significant implications for patient care as it enables the implementation of preventive, empirical, pre-emptive and targeted treatment approaches. However, the presence of newly developed antifungal drugs, particularly triazoles with expanded coverage and improved safety and efficacy, has resulted in a rise in their improper utilization.^4^ Antifungal drugs vary in their potential for interactions with other drugs, clinical effectiveness and safety profiles.^5^ Voriconazole is the first line drug for invasive aspergillosis and is associated with the risk of hepatotoxicity and CNS toxicity. Variability in voriconazole levels is significant due to its nonlinear pharmacokinetics and is influenced by various factors such as patient age, drug metabolism variation due to genetic polymorphism and concurrent medication use.^6^ Previous studies have reported that measuring plasma levels of voriconazole improve patient outcomes and decrease the occurrence of adverse drug reactions.^6^ Amphotericin B is a primary drug for serious fungal infections among paediatric and adult patients but renal insufficiency, hypokalaemia, hypomagnesaemia and metabolic acidosis are major dose limiting side effects.^7,8^ In patients with compromised kidney function, caspofungin is the primary agent for invasive pulmonary aspergillosis and also a preferred agent for candidemia.^9,10^ Caspofungin is well tolerated but may cause infrequent side effects including gastrointestinal disturbance, elevated transaminases and histamine induced reactions.^11^ Research indicates that ∼74% of antifungal drug usage in hospitals may be inappropriate.^12^ Improper utilization of antifungals can lead to negative outcomes, unnecessary exposure leading to increased microbial resistance and health care cost.^5,13^ Therefore, recent clinical guidelines recommend the implementation of antifungal stewardship programmes to address this issue.^14^ Antimicrobial stewardship programmes are designed to ensure proper use of antimicrobial drugs, aiming to reduce the emergence of antimicrobial resistance, lower healthcare costs and decrease rates of morbidity and mortality.^15^ The pharmacist is a key member of multidisciplinary team who can enhance the appropriateness of antifungal prescriptions and quality of antifungal use.^16^ In Pakistan, the data on pharmacist-driven antifungal stewardship activities in oncology setting is lacking although a high proportion of antifungals are used in haemato-oncology departments. The aim of this study was to evaluate the clinical pharmacist-led antifungal drug utilization reviews for optimizing antifungal therapy in a specialized cancer care hospital.

## Patients and method

### Study design and study centre

A pharmacist-led prospective audit and feedback study was conducted at the specialized cancer care hospital of Lahore for 6 months from December 2023 to May 2024. The hospital is dedicated to providing comprehensive cancer care. With a bed capacity of ∼195, Shaukat Khanam memorial cancer hospital and research centre includes specialized departments such as medical and radiation oncology, surgical oncology and paediatric oncology along with the state-of-the-art pharmacy services accredited from Joint Commission International and American Society of Health-System Pharmacists. At this hospital, all patient records are maintained in a computerized hospital information management system (HIMS).

### Prescription review process

**Figure 1.**
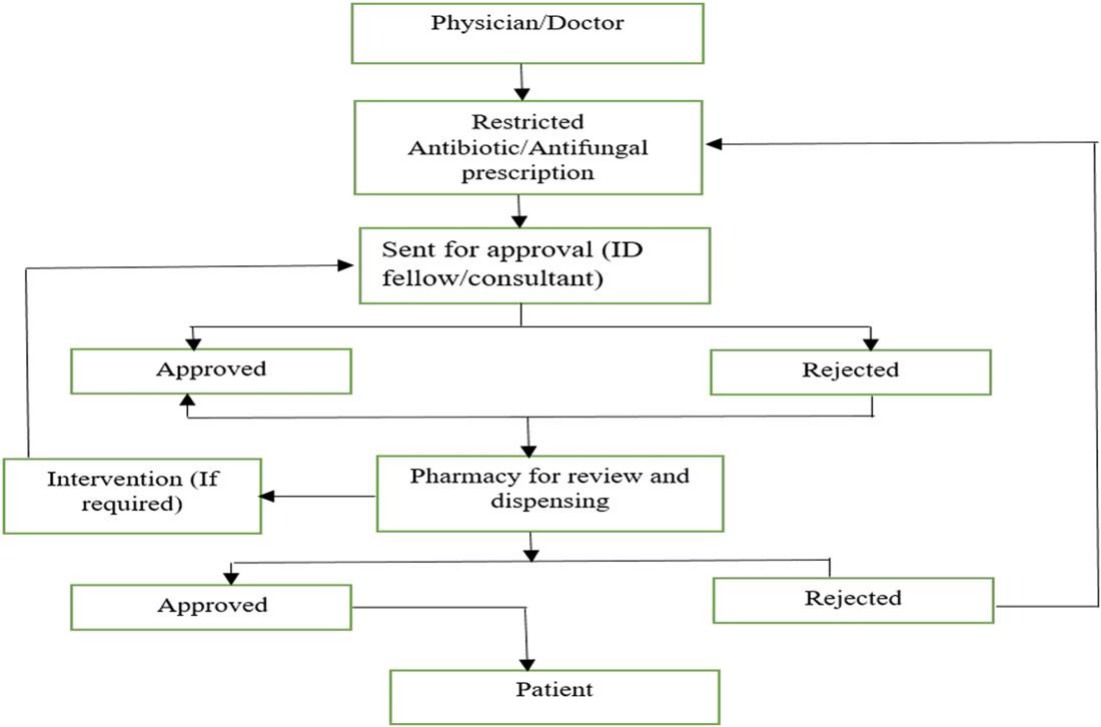
Work flow for restricted antifungals.

Antimicrobial stewardship primarily focusing on antibiotics was implemented in 2012 in the hospital. In 2014, with the introduction of voriconazole as a restricted drug, AFS was initiated. Afterwards, antifungal susceptibility testing was initiated, and a comprehensive AFS programme was fully implemented. An AFS team comprises ID physicians, ID consultants, a microbiologist and an ID clinical pharmacist who are American board certified. They provide pharmaceutical counselling as a part of stewardship regarding selection of antifungals, dose adjustments and drug–drug interactions based on IDSA practice guidelines during ward rounds as well as interpreting the prescriptions on HIMS (pre-prescription authorization and post-prescription review). Physicians enter prescriptions into the system, after which ID clinical pharmacists review them and interventions made during prescription review are documented. Moreover, hospital has also developed guidelines for management of neutropenic patients, thus the acceptability of pharmacist recommendations by AFS team is increased. In case of any discrepancy, decision regarding therapy is made through mutual consensus. Overall work flow for restricted antifungals is shown in Figure 1.

### Study population

Records of all the adult and paediatric patients with cancer who were admitted during December 2023 to May 2024 and received systemic antifungals for >24 hours were included in this study. In case of multiple admissions, the record of first admission was included in the study. Prescription records of repeated patients were excluded.

### Sample size calculation

Sample size was calculated by using the following formula.


$$n=\frac{NZ^{2}p\;(1\;\text{-}\;p)}{d^{2}N+Z^{2}p\;(1\;\text{-}\;p)}$$

The total number of admitted patients (*N*) were 2509 from the years 2019 to 2023 by eliminating repeated admissions. A confidence interval (*Z*) of 95%, margin of error (*d*) 0.05 and antifungal prescription prevalence (*p*) of 50% was incorporated in the sample size formula that yielded 334 records. However, another 16 patients’ data were collected from computerized physician order entry system of the hospital to get a total sample of 350. A systematic sampling technique was used to collect data. The sampling interval was calculated as *K* = *N*/*n* = 2509/350 = 7. Electronic medical records of the admitted patients were generated through the HIMS and every seventh patient was included and reviewed.

### Data collection

A review of the electronic medical record was performed to collect relevant data including patient demographics, type of cancer, type of fungal infection, prescribed antifungal drugs, duration of treatment, radiological tests, biomarkers including beta D glucan and *Aspergillus* galactomannan test, comorbid bacterial infection, concomitant antibiotics used, side effects and pharmacist interventions. Antifungal therapy was classified as empiric, prophylactic, pre-emptive and targeted based on radiological findings, culture results, fungal microscopy and biomarkers.^4,17^

#### Classification of antifungal therapy

***Empiric*** Administered to neutropenic patients experiencing persistent fever that does not respond to 4–7 days of broad-spectrum antibiotics or recurring fever without any symptoms of IFI, in the absence of positive fungal test results. For non-neutropenic patients, treatment is initiated in critically ill individuals who have risk factors for fungal infection and no other identifiable cause for fever.

***Prophylactic*** Initiated for patients who are at high risk of developing IFI, even though they show no signs or symptoms of infection and have negative fungal laboratory test results.

***Pre-emptive*** Targets the early treatment of suspected IFI by using clinical, radiological or laboratory indicators to assess the likelihood of the disease.

***Targeted*** Involves the treatment of confirmed fungal infections based on a positive culture or other diagnostic results.

Side effects observed were classified as elevation of hepatic enzymes: that is, AST, ALT, ALP, elevation of serum bilirubin, serum creatinine and potassium. Therapeutic drug monitoring was available for only voriconazole. First and subsequent levels were documented. Pharmacist interventions are categorized as combination therapy, dose reduction, drug switched to another, drug stopped, dose escalation, voriconazole trough levels missing/inappropriate and IV drug switched to oral.^14^

### Data analysis

Data were entered and analysed using Microsoft Excel and SPSS version 21. Descriptive statistics were applied to determine the frequencies and percentages of categorical variables. Continuous variables were expressed as means.^18^ The Chi-square test was used to identify pharmacist interventions among different antifungal drugs used. Logistic regression was applied to compute the association of independent categorical variables with pharmacist-led interventions. *P* < 0.05 was considered significant.^19^

## Results

### Basic demographics and clinical characteristics of patients

Among 350 patients, 218 (62.3%) were male, 32 (37.7%) were female, 177 (50.6%) were adults, 173 (49.4%) were paediatrics and most (155, 44.3%) patients weighed >45 kg. Among the included patients, most (168, 48.0%) had leukaemia. Febrile neutropenia was present in 194 (55.4%) of the patients. Antifungals were prescribed for suspected fungal infection in most of the patients (146, 41.7%) followed by fungal pneumonia (45, 12.9%) and candidemia (36, 10.3%). The main site of fungal infection was the lungs (140, 40%) as shown in Table 1. Among 136 patients on voriconazole therapy, first voriconazole trough levels were measured in 101 (74.2%) patients. Subsequent second and third trough levels were measured in 43 (31.6%) and 30 (22.1%) patients. Overall, the numbers of patients with low, optimum and high trough levels are shown in Table 1.

**Table 1.** Basic demographics and clinical characteristics of patients

Basic demographics of patients	Frequency *n* = 350	Percentage (%)
Gender		
Male	218	62.3
Female	132	37.7
Age group (years)		
Adult (≥18 years)	177	50.6
Paediatric (<18 years)	173	49.4
Weight (kg)		
<15 kg	93	26.6
16–30 kg	55	15.7
31–45 kg	47	13.4
>45 kg	155	44.3
Type of cancer		
Leukaemia	168	48.0
Lymphoma	80	22.9
Carcinoma	33	9.4
Sarcoma	26	7.4
Others	43	12.3
Febrile neutropenia		
Yes	194	55.4
No	156	44.6
Reason for antifungal prescription		
Suspected fungal infection	146	41.7
Fungal pneumonia	45	12.9
Candidemia	36	10.3
Aspergillosis	25	7.1
Candidiasis	20	5.7
Fungal brain infection	18	5.1
Funguria	18	5.1
Fungal sinusitis	11	3.1
Aspergilloma	10	2.9
Others	13	3.7
Not identified	8	2.3
Site of infection		
Lungs	140	40
Paranasal sinuses	39	11.1
Brain	21	6
Urine	18	5.1
Others	57	16.2
Not defined	75	21.4
Voriconazole first trough levels		
<1.0 µg/mL	31	8.9
1.0–5.5 µg/mL	43	12.3
>5.5 µg/mL	27	7.7
Voriconazole second trough levels		
<1.0 µg/mL	6	1.7
1.0–5.5 µg/mL	17	4.9
>5.5 µg/mL	20	5.7
Voriconazole third trough levels		
<1.0 µg/mL	11	3.1
1.0–5.5 µg/mL	12	3.4
>5.5 µg/mL	7	2.0

### Antifungal drug strategy

Most of the antifungals were prescribed as a targeted therapy (138/350, 39.4%) and empirical therapy (134/350, 38.3%) for IFI. A computed tomography (CT) scan was major positive radiological evidence among 123/350 (35.1%) of patients. Beta D glucan was raised in 73/350 (20.9%) patients and in 29/350 (8.3%) of patients *Aspergillus galactomannan* was raised. Among 350 patients, 428 culture reports were obtained, with blood cultures done in 310 patients (88.5%), urine cultures in 25 (7.14%) patients, tissue cultures in 15 (4.28%) patients, sputum culture in 62(17.7%) patients and wound culture in 16 (4.6%) patients, whereas fungal microscopy was performed in 127 (36.2%) patients. Overall, 92 (26.3%) of the patients had positive blood culture for fungus, while 15 (4.3%), 9 (2.6%), 7 (2.0%) and 6 (1.7%) had positive urine culture, tissue culture, wound culture and sputum culture respectively for fungus. The most frequent isolated fungus was *Candida albicans* (54, 15.4%), followed by *Candida tropicalis* (30, 8.6%) and *Aspergillus flavus* (27, 7.7%) as shown in Table 2.

**Table 2.** Antifungal drug strategy

Antifungal drug strategy	Amphotericin B *n* = 111	Voriconazole *n* = 136	Fluconazole *n* = 27	Caspofungin *n* = 76	Total *n* = 350
Treatment indication					
Targeted	31 (27.9)	43 (31.6)	15 (55.6)	49 (64.5)	138 (39.4)
Empiric	57 (51.4)	38 (27.9)	12 (44.4)	27 (35.5)	134 (38.3)
Pre-emptive	19 (17.1)	45 (33.1)	—	—	64 (18.3)
Prophylactic	4 (3.6)	10 (7.4)	—	—	14 (4.0)
Positive radiological evidence					
CT	40 (36.0)	71 (52.2)	6 (22.2)	7.9	123 (35.1)
XRAY	4 (3.6)	7 (5.1)	—	—	11 (3.1)
BAL	1 (0.9)	6 (4.4)	—	3 (3.9)	10 (2.9)
Positive biomarkers					
Beta D glucan	24 (21.6)	46 (33.8)	—	3 (3.9)	73 (20.9)
*Aspergillus galactomannan*	17 (15.3)	12 (8.8)	—	—	29 (8.3)
Positive cultures					
Blood	10 (9.0)	18 (13.2)	18 (66.7)	46 (60.5)	92 (26.3)
Urine	—	—	3 (11.1)	12 (15.8)	15 (4.3)
Tissue	—	6 (4.4)	—	3 (3.9)	9 (2.6)
Wound	1(0.9)	3 (2.2)	—	3 (3.9)	7 (2.0)
Sputum	—	—	—	6 (7.9)	6 (1.7)
Fungal microscopy	15 (13.5)	10 (7.4)	6 (22.2)	—	31 (8.9)
Isolated pathogen					
*C. albicans*	—	—	6 (22.2)	48 (63.2)	54 (15.4)
*C. tropicalis*	4 (3.6)	13 (9.6)	9 (33.3)	4 (5.3)	30 (8.6)
*A. flavus*	7 (6.3)	20 (14.7)	—	—	27 (7.7)
*C. glabrata*	—	—	—	6 (7.9)	6 (1.7)
*A. fumigatus*	—	5 (3.70)	—	—	5 (1.4)
*C. glabrata*	4 (3.6)	—	—	—	4 (1.1)
*C. krusei*	—	—	—	3 (3.9)	3 (0.9)
*C. parapsillosis*	—	3 (2.2)	—	—	3 (0.9)
*C. pelliculosis*	2 (1.8)	—	—	—	2 (0.6)
*C. neoformans	—	—	6 (22.2)	—	6 (1.7)

*C *=* Candida*, *A *=* Aspergillus*, *C =* Cryptococcus*

### Antifungal prescribing pattern and observed side effects

In most patients, voriconazole use was higher for suspected fungal infections (74/136, 54.4%), fungal pneumonia (26/136, 19.1%) and aspergillosis (22/136, 16.2), however, amphotericin B was used in most patients with fungal sinusitis as compared to voriconazole (9/111, 8.1% versus 2/136, 1.5%) respectively. However, for candidemia, the use of caspofungin was higher (31/76, 48%) as compared to amphotericin B (2/111, 1.8%) and fluconazole (3/27, 11.1%). The average duration of amphotericin B use was 7.3 days, voriconazole 64.1 days, fluconazole 6.8 days and caspofungin 7.9 days. Major side effects observed were hepatotoxicity in 60/350 (17.1%) and hypokalaemia in 47 (13.4%) of the patients followed by elevated serum creatinine 18 (5.1%) and hyperbilirubenimia in 9 (2.6%) patients. Most of the patients (121, 34.6%) were prescribed at least two antibiotics while on antifungal therapy and 51 (14.6%) of the patients were not prescribed any antibiotics as shown in Table 3.

**Table 3.** Antifungal prescribing pattern and observed side effects

Type of fungal infections	Amphotericin B *n* = 111	Voriconazole *n* = 136	Fluconazole *n* = 27	Caspofungin *n* = 76	Total *n* = 350
Suspected fungal infection	57 (51.4)	74 (54.4)	6 (22.2)	9 (11.8)	146 (41.7)
Fungal pneumonia	19 (17.1)	26 (19.1)	—	—	45 (12.9)
Candidemia	2 (1.8)	—	3 (11.1)	31 (40.8)	36 (10.3)
Aspergillosis	10 (9.0)	22 (16.2)	—	3 (3.9)	35 (10.0)
Candidiasis	2 (1.8)	—	6 (22.2)	12 (15.8)	20 (5.7)
Fungal brain infection	—	9 (6.6)	9 (33.3)	—	18 (5.1)
Funguria	—	—	3 (11.1)	15 (19.7)	18 (5.1)
Fungal sinusitis	9 (8.1)	2 (1.5)	—	—	11 (3.1)
Others	7 (6.3)	3 (2.2)	—	3(3.9)	13 (3.7)
Not specified	5 (4.5)	—	—	3(3.9)	8 (2.3)
Average duration of drug use (days)	7.3	64.1	6.8	7.9	29.5
Side effects					
Hepatotoxicity (elevation in AST/ALT)	5 (4.5)	43 (31.6)	6 (22.2)	6 (7.9)	60 (17.1)
Electrolyte imbalance (Hypokalaemia)	39 (35.1)	2 (1.5)	6 (22.2)	—	47 (13.4)
Nephrotoxicity (Elevated serum creatinine)	13 (11.7)	2 (1.5)	—	3 (3.9)	18(5.1)
Hyperbilirubenimia	1(0.9)	5 (3.7)	3 (11.1)	—	9 (2.6)
Concomitant antibiotic usage (no.)					
1	16 (14.4)	29 (21.3)	6 (22.2)	10 (13.2)	61 (17.4)
2	49 (44.1)	44 (32.4)	13 (48.1)	15 (19.7)	121 (34.6)
3	16 (14.4)	35 (25.7)	5 (18.5)	33 (43.4)	89 (25.4)
4	5 (4.5)	14 (10.3)	—	9 (11.8)	28 (8.0)
Antibiotics not prescribed	25 (22.5)	14 (10.3)	3 (11.1)	9 (11.8)	51 (14.6)

### Pharmacist interventions related to antifungal therapy

Among 350 patients, 272 (77.71%), interventions in therapy were made by pharmacists. Most interventions were related to switching of intravenous amphotericin B, voriconazole and caspofungin to oral voriconazole (63/350, 18.0%), choice in the selection of a drug (61/350, 17.4%), dose reduction (59/350, 16.9), drug stopped (41/350, 11.7%) and dose escalation (37/350, 10.6%). All the interventions made by pharmacists were accepted by physicians (100%). However, no interventions were made in prescriptions of 78 (22.3%) patients as shown in Table 4.

**Table 4.** Pharmacist interventions drug wise

Interventions	Amphotericin B *n* = 111	Voriconazole *n* = 136	Fluconazole *n* = 27	Caspofungin *n* = 76	Total *n* = 350	*P* value
IV switched to oral azole	49 (44.1)	11 (8.1)	3 (11.1)	—	63(18.0)	0.00***
Drug switched to another/choice of a drug therapy	14 (12.6)	2 (1.5)	3 (11.1)	42 (55.3)	61(17.4)	
Dose reduction	5 (4.5)	43 (72.9)	1 (3.7)	10 (13.2)	59(16.9)	
Drug stopped	6 (5.4)	23 (16.9)	6 (22.2)	6 (7.9)	41(11.7)	
Dose escalation	4 (3.6)	30 (22.1)	3 (11.1)	—	37(10.6)	
Combination therapy	2 (1.8)	4 (2.9)	5 (18.5)	—	11 (3.1)	
No intervention	31 (27.9)	23 (16.9)	6 (22.2)	18 (23.7)	78 (22.3)	
Interventions accepted by AFS team	80 (29.4)	113 (41.5)	21 (7.7)	58 (21.3)	272 (100)	

*significant at *P* < 0.1, **significant at *P* < 0.05, ***significant at *P* < 0.01, (−) indicates no intervention

### Association of pharmacist intervention with independent variables

Effect of gender, age group, type of cancer, type of antifungal therapy and therapeutic indication on pharmacist interventions were compared as shown Table 5. In univariate logistic regression, gender, age group and type of antifungal do not significantly affect the likelihood of pharmacist interventions (*P* > 0.05). However, pharmacist interventions were significantly higher when antifungal therapy was prescribed pre-emptively (*P *= 0.01, OR; 3.7) compared to the reference category, i.e. empirical use and significantly lower in patients diagnosed with carcinoma (*P *= 0.07) (OR; 0.4) compared to the reference group, i.e. lymphoma as shown in Table 5. Multivariate logistic regression revealed pharmacist interventions were significantly less likely in paediatric patients (OR 0.4), patients with carcinoma (OR 0.2) and some other types of cancer (OR 0.4). However, pharmacist interventions were significantly higher in patients prescribed with fluconazole (*P *= 0.05) and caspofungin (*P *= 0.01) (OR 3.0) and in the pre-emptive group (*P *= 0.03) (OR 3.2) as shown in Table 5.

**Table 5.** Logistic regression analysis of covariates on pharmacist intervention

Covariates	Pharmacist interventions	Unadjusted odds ratio		Adjusted odds ratio	
		OR (95% CI)	*P* value	OR (95% CI)	*P* value
Gender					
Female	102 (29.1)	Reference		Reference	
Male	170 (48.5)	1.0 (0.6–1.7)	0.87	0.8 (0.4–1.4)	0.51
Age group					
Adult	141 (40.2)	Reference		Reference	
Paediatric	131 (37.4)	0.7 (0.4–1.3)	0.37	0.4 (0.2–0.8)	0.01**
Type of cancer					
Lymphoma	139 (51.1)	Reference		Reference	
Leukaemia	64 (23.5)	1.1 (0.6–2.3)	0.60	1.6 (0.7–3.7)	0.19
Carcinoma	21 (7.7)	0.4 (0.2–1.1)	0.07*	0.2 (0.0–0.6)	0.006***
Sarcoma	19 (6.9)	0.7 (0.2–1.8)	0.45	1.1 (0.3–3.3)	0.87
Others	29 (10.6)	0.5 (0.2–1.2)	0.12	0.4 (0.1–1.0)	0.05*
Antifungal drug					
Amphotericin B	80 (22.8)	Reference		Reference	
Voriconazole	113 (32.2)	0.8 (0.4–1.5)	0.51	1.7 (0.9–3.3)	0.10
Fluconazole	21 (6.0)	1.5 (0.7–3.1)	0.23	3.0 (0.9–9.2)	0.05*
Caspofungin	58 (16.6)	1.0 (0.3–3.1)	0.87	3.0 (1.2–7.6)	0.01**
Indication					
Empiric	102 (29.14)	Reference		Reference	
Targeted	99 (28.2)	0.8 (0.4–1.3)	0.41	0.8 (0.4–1.5)	0.59
Pre-emptive	59 (16.9)	3.7 (1.3–10.0)	0.01**	3.2 (1.1–9.5)	0.03**
Prophylactic	12 (3.42)	1.8 (0.4–8.8)	0.42	1.0 (0.2–5.4)	0.96

*significant at *P* < 0.1, **significant at *P* < 0.05, ***significant at *P* < 0.01

## Discussion

The aim of this study was to investigate clinical pharmacist-led antifungal drug utilization reviews for optimizing antifungal therapy in patients with cancer. Previously published studies in Pakistan have focused on antibacterial stewardship and the role of pharmacists in optimizing antibacterial use.^20^ To our knowledge, this is the first study from Pakistan evaluating pharmacist-led antifungal drug utilization reviews. Results of this study showed that most patients had lymphoma (22.9%) and leukaemia (48.0%). These findings were consistent with a previous study that showed haematological malignancy is a major risk factor for systemic fungal infections.^4^ The most common site of infection was the lungs as reported previously.^21^ More than half of patients (55.4%) had febrile neutropenia similar to a previous study that showed 64.8% patients with IFD had febrile neutropenia.^22^ Of the culture test performed, the most frequently isolated pathogen was *C. albicans* (15.4%), followed by *C. tropicalis* (8.6%) and *A. flavus* (7.7%). A study conducted in 2013 in similar setting reported highest prevalence of *C. tropicalis* (30.8%) compared to *C. albicans* (3.3%).^23^ The literature report the highest prevalence of *C. tropicalis* followed by *C. albicans* among positive isolates in patients with haematological malignancies (41.1% versus 28.6%).^24^ Our results are consistent with a previous study conducted in a tertiary care hospital in Brazil that showed the most isolated microorganism was *C. albicans* (36.5%).^25^ Another study conducted in Iran reported the highest prevalence of *A. flavus*.^26^ Differences in isolated pathogen frequencies reflect geographical variation and population differences and diagnostic techniques.

Most patients with cancer in this study were prescribed systemic antifungals for suspected fungal infections leading to increased empirical consumption of antifungals. Our results showed almost similar utilization of targeted and empirical antifungal therapy. These findings were different from previous literature that reported higher prophylactic use of antifungals.^4,27^ Guidelines also recommend the use of empirical AFT in patients who remained febrile 4–6 days after using broad-spectrum antibiotics.^2^ These findings reflect adherence to treatment guidelines in the observed setting. A study conducted by Kaur. H *et al.,* reported higher prescription of targeted antifungal therapy compared to empirical therapy.^28^ According to results, voriconazole is mostly prescribed for fungal pneumonia (19.1%) and aspergillosis (16.2%), for candidemia and candidiasis, caspofungin was used in most patients (40.8%) and for fungal sinusitis (8.1%), amphotericin B was prescribed mostly. For candidemia and invasive candidiasis, the literature showed that 71.1% patients develop successful overall response with caspofungin.^29^ For aspergillosis, voriconazole is recommended as the initial drug therapy.^5,6^ The results of a previous study also reported better outcomes with amphotericin B in fungal sinusitis.^30^

The most frequently observed side effects with voriconazole and fluconazole were elevation in AST/ALT and hyperbilirubenimia, whereas nephrotoxicity with amphotericin B was similar to that previously reported.^5,31^ These side effects necessitate pharmacist-led interventions regarding change in therapy and dose adjustments thus optimizing therapy. Most patients on voriconazole who undergo therapeutic drug monitoring had their plasma trough concentration in optimum range (1.0–5.5 µg/mL) with 43% patients achieving targeted concentration with initial dosing compared to 50% reported previously.^32,33^ However, plasma trough concentrations were supra-therapeutic in 7.7% patients and sub-therapeutic in 22.8% patients, reflecting the need for subsequent level monitoring of voriconazole because it has nonlinear pharmacokinetics.^34^ In this hospital, the ID pharmacist adjusts the dose of voriconazole based on plasma levels to ensure optimal efficacy and minimize toxicity.

Our findings showed that almost 77.7% interventions were made by the ID clinical pharmacist and all the interventions were accepted by the AFS team. Major interventions were related to switching from IV therapy to oral (18%) and choice in the selection of antifungals (17.4%), so that when voriconazole and amphotericin B cannot be prescribed because of hepatotoxicity and nephrotoxicity and dose reduction (16.9%). However, in a previous study, a recommendation regarding selection of a drug was made in only 1.3% of patients, dose reduction in 6.54% of patients and administration route-related recommendations in 3.8% patients.^14^ Furthermore, when the ID pharmacist identifies drug–drug interactions, the medication may be either discontinued or substituted. In some cases, dose adjustments, such as escalation or reduction, are implemented to manage the interaction effectively. These interventions result in improved patient safety and optimized therapy. In this study, pharmacist recommendations were significantly higher in the pre-emptive therapeutic approach (*P *= 0.01). Because pre-emptive therapy starts early before symptoms of the disease fully develop, pharmacists intervene by adjusting dosages, identifying potential drug interactions and recommending adjunct therapies to manage or prevent adverse effects. This might have resulted in higher number of interventions in the pre-emptive group. It has been previously reported that implementation of a pharmacist review increased the appropriate use of antifungal prophylaxis guidelines from 31% to 54%.^3^ Another study’s results reflected that pharmacist-driven AFS recommendations led to a significant improvement in dosage accuracy (*P* < 0.05) and the correct selection of antifungal drugs (*P* < 0.05), along with a reduction in potential clinically relevant drug–drug interactions with concomitant medications (*P* < 0.05).^18^ Our results also showed that the clinical pharmacists’ involvement in the AFS team enhanced the optimum utilization of antifungal agents within the examined hospital setting. Pre-prescription authorization, post-prescription review and feedback constitute the fundamental elements of stewardship.^14^ Therefore, pharmacist-led interventions have the potential to enhance the appropriateness of antifungal treatments and improve patient safety. It is essential to have clinical pharmacists as an active part of the patient health care team to optimize the antifungal treatment, especially from the pharmacokinetic perspective, as it is the domain in which pharmacist can perform better than other health care professionals. Pharmaceutical care should be encouraged in low–middle-income countries where health resource constraints lead to suboptimal care of patients.

### Study limitations

This study has a few limitations. First, it was conducted in cancer care hospital focusing only on patients with cancer who had systemic fungal infections. Second, the effects of pharmacists’ interventions on mortality and length of hospital stay cannot be ascertained because of the underlying disease related challenges in distinguishing mortality and length of hospital stay.

### Future investigation

Future research can investigate the impact of pharmacist recommendations regarding antifungal therapy on economic outcomes by collecting data from different hospitals. This approach will provide a more comprehensive understanding of the economic benefits associated with pharmacists’ contributions to patient care.

### Conclusion

The prevalence of systemic fungal infections was notably higher in patients with leukaemia, necessitating robust antifungal management. Most antifungal prescriptions were administered as targeted and empirical therapy, with fungal pneumonia being the primary indication for treatment. Voriconazole was commonly used with TDM to maintain optimal drug levels, resulting in improved patient outcomes and minimized toxicity. Additionally, the pharmacist-led interventions were primarily focused on optimizing the administration route, selecting the appropriate antifungal and adjusting doses, which significantly enhanced the quality and safety of antifungal prescribing. Key interventions included regular electrolyte monitoring with amphotericin B to manage common adverse effects such as hypokalaemia and hypomagnesaemia. For voriconazole, the TDM process was supplemented with drug interaction monitoring, given its high potential for interactions, ensuring safe and effective drug levels. Notably, all clinical pharmacist recommendations were accepted by the prescribing physicians, demonstrating the value of collaborative antifungal stewardship in enhancing patient care and supporting optimal antifungal therapy in oncology settings.

## Data Availability

All the data gathered and analysed are available in the article.
